# Zero-bias mid-infrared graphene photodetectors with bulk photoresponse and calibration-free polarization detection

**DOI:** 10.1038/s41467-020-20115-1

**Published:** 2020-12-17

**Authors:** Jingxuan Wei, Ying Li, Lin Wang, Wugang Liao, Bowei Dong, Cheng Xu, Chunxiang Zhu, Kah-Wee Ang, Cheng-Wei Qiu, Chengkuo Lee

**Affiliations:** 1 Department of Electrical and Computer Engineering, National University of Singapore, Singapore, 117583 Singapore; 2 Center for Intelligent Sensors and MEMS, National University of Singapore, Singapore, 117608 Singapore; 3 Interdisciplinary Center for Quantum Information, State Key Laboratory of Modern Optical Instrumentation, College of Information Science and Electronic Engineering, Zhejiang University, Hangzhou, 310027 China

**Keywords:** Optical properties and devices, Mid-infrared photonics, Optoelectronic devices and components, Metamaterials, Imaging and sensing

## Abstract

Bulk photovoltaic effect (BPVE), featuring polarization-dependent uniform photoresponse at zero external bias, holds potential for exceeding the Shockley-Queisser limit in the efficiency of existing opto-electronic devices. However, the implementation of BPVE has been limited to the naturally existing materials with broken inversion symmetry, such as ferroelectrics, which suffer low efficiencies. Here, we propose metasurface-mediated graphene photodetectors with cascaded polarization-sensitive photoresponse under uniform illumination, mimicking an artificial BPVE. With the assistance of non-centrosymmetric metallic nanoantennas, the hot photocarriers in graphene gain a momentum upon their excitation and form a shift current which is nonlocal and directional. Thereafter, we demonstrate zero-bias uncooled mid-infrared photodetectors with three orders higher responsivity than conventional BPVE and a noise equivalent power of 0.12 nW Hz^−1/2^. Besides, we observe a vectorial photoresponse which allows us to detect the polarization angle of incident light with a single device. Our strategy opens up alternative possibilities for scalable, low-cost, multifunctional infrared photodetectors.

## Abstract (web summary)

Here, graphene-based plasmonic metamaterials are used to generate an artificial bulk photovoltaic effect, enabling the realization of mid-infrared photodetectors with enhanced responsivity and calibration-free polarization detection at room temperature.

## Introduction

Optoelectronic devices play critical roles in widespread applications, including imaging, communication, sensing, and energy harvesting^1,2^. A recent technological advance in this field is the bulk photovoltaic effect (BPVE)^3–6^. Unlike the conventional photovoltaic and photoconducting effects, the BPVE does not require a junction and provides a bandgap-unlimited photovoltage under uniform illumination, holding the potential for exceeding the Shockley–Queisser limit^7^. The primary mechanism of this effect is the shift current, where the excited photoelectrons have intrinsic momenta and are shifted in real space upon interband optical transitions^8–10^. As a second-order nonlinear optical process, the shift current can only be observed in materials with broken inversion symmetry, such as ferroelectrics^11^. Due to the spontaneous polarization in such materials, the BPVE usually shows a clear dependence on the polarization states of illumination. These properties of the BPVE have motivated great interests in the designs for high-efficiency functional photodetectors. However, the practical implementation of the BPVE is still hindered by several problems. First of all, the available materials for the BPVE are primarily limited in ferroelectrics and the emerging topological materials, which suffer from low efficiencies and fabrication difficulties^1,3,12,13^. Although recent studies suggest that larger BPVE may exist in low-dimensional materials, experimental demonstrations are still rare^5,9^. One alternative approach could be the flexo-photovoltaic effect, in which strain gradients are applied to introduce broken inversion symmetry in the materials even without intrinsic BPVE^6,14^. However, it remains challenging for this approach to fabricate large-scale devices. Second, the existing devices mainly work in the visible range^3,5,6^. However, efficient infrared detection is also highly desired with widespread applications such as solar energy harvesting, free-space communication, environmental monitoring, and thermal imaging^15–20^. Particularly, it would be of great interest if the BPVE can offer possibilities for uncooled infrared photodetectors with high efficiency and low cost, which remains as one of the major challenges for the current infrared technologies^1,16^.

Metamaterials, the artificial materials with sub-wavelength structures and highly engineered optical properties, offer unique opportunities for optoelectronic devices^21–30^. Due to the plasmon resonance, the near-field intensity and hence the light absorption can be enhanced by several orders^31–33^. Besides, the generated photocarriers may be efficiently collected by the metallic sub-wavelength structures, which can act as electrodes^34^. Importantly, the exotic properties of metamaterials will equip the devices with functionalities such as the dependence on polarization angle^34,35^, handedness^36^, angle of incidence^27^, and wavelength^24,37^. To date, metamaterials have been widely used to enhance the conventional mechanisms including photovoltaic^37^, photo-thermoelectric^38^, photoconducting^39^, and pyroelectric effects^22^. However, to the best of our knowledge, metamaterials have not been experimentally exploited for the BPVE^40^.

In this work, we present a paradigm for metasurface-mediated graphene photodetectors with BPVE-type features. Since the bulk response in our devices originates from the non-centrosymmetric sub-wavelength structure of plasmonic nanoantennas rather than materials, we refer to this effect as an artificial BPVE. Our simulation and experiment suggest that the non-centrosymmetric nanoantennas break the symmetry of local field and help photocarriers to gain a momentum via the gradient of Seebeck coefficient and conductive guidance. As a proof of the usefulness of our design, we have demonstrated an uncooled mid-infrared photodetector with zero stand-by power consumption, with a measured noise equivalent power as low as 0.12 nW Hz^−1/2^, which is competing with the commercial devices that require external bias. Furthermore, we observe vectorial photocurrents in the artificial BPVE, which can be used for unambiguous detection of polarization angles with a single device regardless of the incident power.

## Results

### Metasurface-mediated graphene photodetectors design

**Fig. 1:**
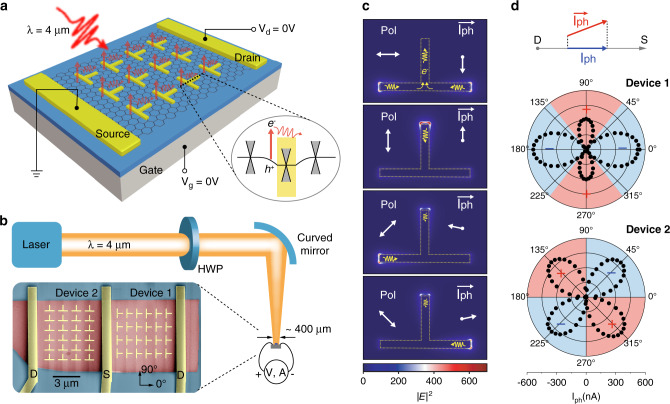
Design concept and main results of metasurface-mediated graphene photodetectors. **a** Illustration of the designed metasurface-mediated graphene photodetector, which consists of non-centrosymmetric sub-wavelength metallic nanoantennas as meta-atoms on top of graphene flakes. Under uniform illumination at 4 µm wavelength, global directional photocurrents are generated from each meta-atom at zero external bias (*V*_d_ = *V*_g_ = 0 V), mimicking the shift current in the bulk photovoltaic effect (BPVE). Importantly, due to the gapless nature of graphene, the local photoresponse from nanoantennas can efficiently contribute to the external circuit in a nonlocal manner, enabling a cascaded total photocurrent. From bottom to top: Si (gray), SiO_2_ (blue), graphene (black honeycomb), Pd/Au for nanoantennas, and electrodes (yellow). Inset illustrates the excitation of electrons at one edge of nanoantennas and the following directional transport. Black line denotes the band diagram of graphene. Yellow area is the graphene region that is covered and doped by metal. **b** Schematic of the experimental setup with control of the linear polarization state via rotation of half-wave plate (HWP). The focused laser beam has a beam diameter around 400 μm, which is much larger than the size of our device. The inset shows the scanning electron microscopy (SEM) image of our device in false colors: graphene in dark red, nanoantennas, and electrodes in yellow, substrate in dark blue. **c** Simulated near-field distribution and predicted vectorial photocurrent in a unit cell at different polarization angles of incident light (Pol). |*E*|^2^ represents the intensity of local electrical field. Yellow wave arrows indicate the flow of photocarriers generated at the nanoantenna–graphene interfaces. White arrows illustrate the resultant vectorial photocurrents (***I***_***ph***_). **d** Polar plot of measured *I*_ph_, which is the scalar projection of ***I***_***ph***_ on the orientation of drain–source electrodes. Red and blue areas mark the positive and negative signs of ***I***_***ph***_.

A schematic perspective view of the designed metasurface-mediated photodetector is sketched in Fig. 1a. The metasurface consists of non-centrosymmetric metallic nanoantennas as meta-atoms on top of graphene flakes which are exfoliated on silicon wafers with 285 nm thermal oxide. A back gate is used to tune the doping level and hence Fermi level of graphene for mechanism studies. Under uniform illumination and at zero external bias (*V*_d_ = *V*_g_ = 0 V), the generated photocarriers are shifted in real space with the direction and magnitude of the shift currents controlled by the polarization of light.

The fabricated devices are characterized with a quantum cascaded laser at 4 μm wavelength as shown in Fig. 1b, with the scanning electron microscopy (SEM) image as the inset. The polarization states of the incident light are linear, with their angles controlled via rotation of a half-wave plate (HWP). A curved mirror is used to deflect and focus the light, and the beam diameter on the device plane is several hundreds of micrometers (Supplementary Note 8). Two devices with 6 by 4 array of T-shaped nanoantennas are fabricated side-by-side on a few-layer graphene flake. The orientation of the nanoantennas in Device 1 is rotated by 90° compared to Device 2. The T-shaped nanoantennas are designed to have strong resonance at 4 μm (see Supplementary Note 2). The vertical and horizontal lengths of the nanoantenna unit in Device 1 are 1.1 and 0.6 μm, respectively. The respective vertical and horizontal pitches are 1.5 and 1 μm, which are smaller than half of the wavelength. The distance between neighboring nanoantennas is kept above 300 nm to avoid mutual near-field coupling to simplify our design. The composition of both nanoantennas and electrodes is 5 nm palladium as contact layer and 50 nm gold on top. The measured Raman spectra and atomic force microscopy images can be found in Supplementary Notes 3 and 4. Because the device areas are much smaller than the beam size, we can regard the illumination on the device to be uniform.

The mechanism of our device is explained as follows. It has been widely accepted that hot electron mechanism dominates the photoresponse in graphene^41–44^. In this work, we consider the photocurrents to originate from local heating of light and subsequent heat flow via non-uniformities in Seebeck coefficient, *S*, with the direction and amplitude determined by the gradient of *S*, that is, ${\boldsymbol{I}}_{{{{\mathbf{ph}}}}} \propto \left| E \right|^2 \cdot \vec \nabla S.E$ is the amplitude of light at the local area. In our design, the Seebeck coefficient gradient is formed by depositing isolated metallic nanoantennas directly on the surface of graphene, where the graphene covered by metal will be doped^45^. The direction of gradient is then along the normal of the metal-graphene interfaces, which points inward or outward depending on the relative Seebeck coefficients of graphene underneath and out of the metal^46^ (see Supplementary Note 1 for more discussion on the Seebeck coefficient of graphene). Since the edges of these isolated nanoantennas are closed, the overall integrated photocurrents along the edge of nanoantenna under uniform illumination should be simply negligible, ${\boldsymbol{I}}_{{{{\mathbf{ph}}}}} \propto \left| E \right|^2 \cdot \left| {\vec \nabla S} \right| \cdot {\oint}_l {\vec ndl \approx 0}$. To achieve non-zero photocurrents, the uniformity of illumination can be broken with focused light^44,47^, which is, however, more challenging and less useful for practical applications. To address this bottleneck, we design the shape of nanoantennas as non-centrosymmetric to create large asymmetry in the local field via plasmon localization effect. Considering a perfect case where all the light is focused to a certain point, the resultant photocurrent would be ${\boldsymbol{I}}_{{{{\mathbf{ph}}}}} \propto \left| {E_{{\mathrm{enhanced}}}} \right|^2 \cdot \left| {\nabla S} \right|{\oint}_l {\delta \left( l \right) \cdot {\boldsymbol{n}}dl = \left| {E_{{\mathrm{enhanced}}}} \right|^2 \cdot \left| {\nabla S} \right| \cdot } {\boldsymbol{n}}_0$, which is unbalanced, enhanced, and directional. ***n***_**0**_ is the normal vector of the metal-graphene interface at that point. *E*_enhanced_ is the plasmonic enhanced local field, which can be several orders higher than *E*. In the discussion above, we have assumed that the photocurrent behaves in a ballistic manner. In reality, the mean free path of carriers in graphene are within a few micrometers at room temperature^48^, so that diffusive transport must also be considered as suggested by our experiments. Besides, due to the semimetal nature of graphene, the photocurrent is not only directional but also global, which can be regarded as the collective movement of charge carriers with a momentum as captured by a Shockley–Ramo-type framework^49^.

Although we cannot provide a quantitative framework at this stage, we intuitively propose two possible ways for the photocarriers to gain their momentum, with the numerical analysis and experimental results shown in Fig. 1c, d, respectively. The first way is rather straightforward, that is, the photocarriers can be driven via the gradient of Seebeck coefficient, in which we believe ballistic transport plays a dominant role^48^. Besides, the photocarriers could also gain a momentum via the guidance of high conductance of metallic nanoantennas, for which diffusive transport is responsible. The supporting evidence for our claim as follows. In the case of Device 1 at 90° polarization angle, although our simulation shows that the local illumination is symmetric and should forbid a net photocurrent in perfect ballistic case, we do have observed large positive photocurrent. This can only be explained by the diffusive transport in the effect of asymmetric guidance of the horizontal bar of nanoantennas in Device 1, as shown in the top sub-graph of Fig. 1c. On the other hand, the diffusive transport alone cannot explain why the photocurrent of Device 1 at 0° polarization angle is as two times large as that at 90° polarization angle. In fact, we have repeatedly observed that the normal of illuminated graphene-metal interfaces should point to the drain–source direction to achieve maximum photoresponse. This possibly indicates that photocarriers can gain larger momenta in the ballistic case than the diffusive case. The sign-flipped photocurrents in Device 2 at 45° and 135° polarization angles can also be well explained by the near-field analysis.

Although the proposed explanation above is still relatively qualitative, our near-field simulation and experiments reinforce each other in good agreement. It is worth noting that the alignment of nanoantennas in Device 2 is rotated by 90° compared to Device 1, but the polarization pattern is only rotated by 45° and the positive and negative photocurrents are almost equal in amplitude. Besides, the polarization controlled sign flipping of photocurrents are unusual in previous photodetectors with intrinsic anisotropy^50,51^. This occurs because of the vectorial nature of photocurrents in our design. In experiments, we can only measure the scalar projection of the ***I***_**ph**_ on the orientation of drain–source electrodes. By rotating the nanoantennas pattern by 90° from Device 1 to Device 2, we measure the other component of ***I***_**ph**_ projected on the orthogonal directions, rather than a trivial repeated measurement. Based on all the evidence above, we conclude that the nanoantennas help the photocarriers to gain a momentum, which features a vectorial nature of photocurrent, ***I***_**ph**_.

Following the convention of the BPVE^9^, the photocurrent in our two-dimensional devices can be written as

1 $$\begin{array}{*{20}{c}} {I_{{\mathrm{ph}},1} = \mathop {\sum }\limits_{i,j} \alpha _{ij}E_i^ \ast E_j} \end{array}$$

2 $$\begin{array}{*{20}{c}} {I_{{\mathrm{ph}},2} = \mathop {\sum }\limits_{i,j} \beta _{ij}E_i^ \ast E_j} \end{array}$$

where *i* and *j* denote the polarization directions *x* or *y*. The *α*_*ij*_ and *β*_*ij*_ are the components of second-rank tensors

3 $$\begin{array}{*{20}{c}} {\alpha = \left( {\begin{array}{*{20}{c}} {\alpha _{xx}} & {\alpha _{xy}} \\ {\alpha _{yx}} & {\alpha _{yy}} \end{array}} \right)\sim \left( {\begin{array}{*{20}{c}} { - 1.1} & 0 \\ 0 & {0.6} \end{array}} \right)} \end{array}$$

4 $$\begin{array}{*{20}{c}} {\beta = \left( {\begin{array}{*{20}{c}} {\beta _{xx}} & {\beta _{xy}} \\ {\beta _{yx}} & {\beta _{yy}} \end{array}} \right)\sim \left( {\begin{array}{*{20}{c}} 0 & { - 1} \\ 1 & 0 \end{array}} \right)} \end{array}$$

The values in the matrix are extracted from the experimental results.

### Validation of bulk photoresponse

**Fig. 2:**
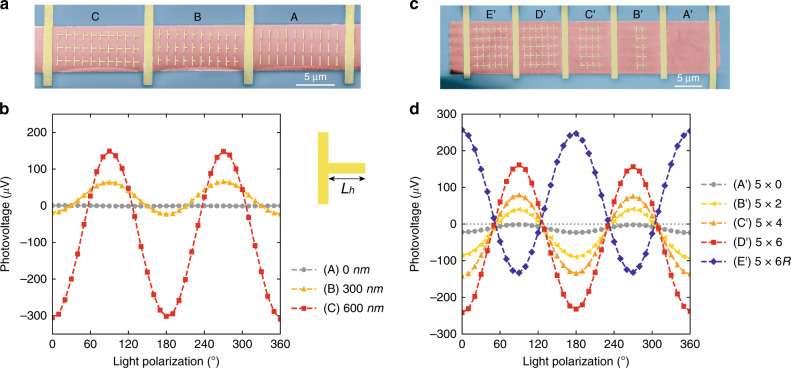
Evidence of bulk photoresponse. **a**, **b** SEM images and measured photovoltage of three devices with different degrees of asymmetry. When the lengths of horizontal arms (*L*_h_) decrease from device *C* to *A*, the photoresponse decreases and vanishes eventually due to the absence of broken symmetry. **c**, **d** SEM images and measured photovoltage of five devices with different numbers of nanoantennas, where the nanoantennas closest to the contact electrodes are removed gradually from Device *D*′ to *A*′. Device *E*′ has the same number of nanoantennas as device *D*′, but the orientation of nanoantennas is reversed. The photovoltages is only dependent on the number of nanoantennas but insensitive to their position. The non-zero photoresponse of device *A*′ could be due to non-uniform illumination, fabrication imperfections or nonlocal photoresponse from neighboring devices. Error bars are smaller than the size of markers. Dashed lines are guides to the eye.

To further validate the metasurface-mediated BPVE-type photoresponse, we have designed two groups of control experiments to investigate the effect of symmetry, orientation and number of nanoantennas. To have a fair comparison, the devices in the same group have been fabricated through the same processes and with the same channel length and width. As shown in Fig. 2a, b, when the nanoantennas are more symmetric with decreasing lengths of the horizontal arm, *L*_h_, the BPVE-type features become less observable and disappear eventually. This occurs because the symmetric nanoantennas fail to provide a net momentum to the excited photocarriers through either Seebeck effect or metallic guidance (see Supplementary Note 5 for near-field analysis). Furthermore, it is also critical to validate the cascaded position-insensitive photoresponse in our device as predicted by the Shockley–Ramo framework^49^. As shown in Fig. 2c, we have fabricated devices with decreasing column numbers of nanoantennas from 6 in Device *D*′ to 0 in Device *A*′. Importantly, the nanoantennas are removed gradually from the edge of array which are closest to the contact electrodes, so that we can examine the possible position dependent photoresponse. Figure 2d shows the measured results, where the photocurrent is almost linear to the number of nanoantennas (see Supplementary Note 6 for the linear fitting). This indicates that the photoresponse of nanoantennas is global and position insensitive. Besides, we have designed Device *E*′ with the same column and row numbers as device *D’* but a reversed orientation. Unsurprisingly, we observe reversed photovoltage when the orientation of nanoantennas is flipped. Although the above evidences are sufficiently convincing to support our claim, we also observe some deviation from our prediction in Device *A*′. The small nonzero photoresponse in Device *A*′ could be due to nonuniform illumination, fabrication imperfection or nonlocal photoresponse from neighboring devices due to plasmonic heating induced thermal gradient^46^. We also note that the error bars in Fig. 2b, d are smaller than the size of markers, since we have used a lock-in amplifier to significantly reduce the noise level with the incident light modulated by an optical chopper. Overall, we can conclude the BPVE-type photocurrent to be generated from the non-centrosymmetric nanoantennas instead of the contact or other effects. Besides, it is also confirmed that the nanoantenna-assisted photoresponse is position-insensitive and hence can be conveniently cascaded.

### Device characterization

**Fig. 3:**
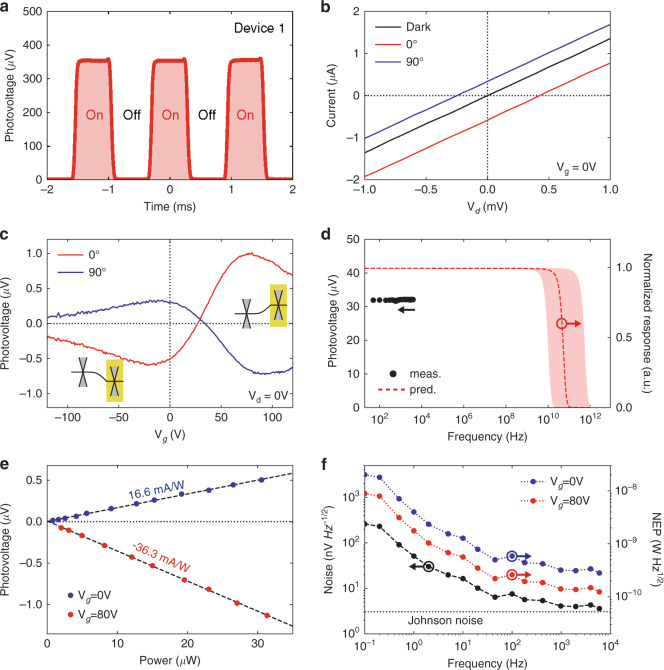
Device characterization. **a** Measured photovoltage during on-off cycles of an incident laser at 4 μm wavelength. Light is modulated with an optical chopper at 800 Hz. **b** Measured currents versus applied *V*_d_ at dark and light conditions, with their difference as the photocurrents. 0° and 90° denote the polarization angles of the incident light. The intersections with *x* and *y* axes represent open-circuit voltage and short-circuit current, respectively. **c** Measured photocurrent versus gate voltage at 0° and 90° polarization angles. Inset shows the respective band diagrams. While the Fermi level of the graphene covered by nanoantennas (yellow region) is pinned, the Fermi level of uncovered graphene channel can be tuned by gate voltage, leading to flipping of band bending and hence opposite photoresponse. **d** Measured and predicted frequency response. Red shaded area denotes the range of reported data and hence the uncertainty of our estimation. **e** Dependence of photocurrents on the power of incident light at two different gate voltages. The polarization angle is 0°. **f** Measured frequency-dependent noises and calculated noise equivalent power (NEP). The similar trend between noise and NEP is due to the almost constant frequency responsivity. Johnson noise becomes dominant for frequencies above 1 kHz.

After verifying the metasurface-mediated BPVE-type photoresponse in our device, we further characterize Device 1 in terms of *I*–*V* characteristics, responsivity, noise, response speed, and gate tuning. Figure 3a illustrates the time response with the incident light modulated by an optical chopper at 800 Hz frequency. The rise and fall times are about 100 μs which are limited by the chopping speed. Figure 3b displays the *I*–*V*_d_ characteristics at zero gate voltage under dark and illuminated conditions, in which 0° and 90° represent the polarization angles of incident light. The linear *I*–*V*_d_ lines indicate the Ohmic contact between metal and graphene. Under illumination, the *I*–*V*_d_ lines are shifted from the origin, with two intersections on *x* and *y* axes referred to as open-circuit voltage and short-circuit current, respectively. Besides, the *I*–*V*_d_ lines are shifted toward different sides for 0° and 90^o^ polarization angles, illustrating their opposite photoresponse. We also investigate the gate dependence of the short-circuit current as Fig. 3c. During the forward sweeping of gate voltage, we find two photocurrent peaks with opposite signs around 0 and 80 V, while zero is achieved at *V*_g_ = 35 V where flat band condition is fulfilled. The measured non-monotonous *I*–*V*_g_ curves are typical in the graphene–metal contact interfaces, confirming the origin of dominant photocurrent as the hot carrier-assisted photo-thermoelectric effect^37,41,43^. The applied gate voltage can electrically dope the graphene, and hence tune the Seebeck coefficient gradient and photocurrent direction as the insets^46,52,53^. We also note that the peak photocurrent around *V*_g_ = 80 V is almost twice as that around *V*_g_ = 0 V. This occurs because the metal Pd dopes graphene as *p*-type, and hence *p*–*n* junctions are formed at *V*_g_ = 80 V but *p*–*p*′ junctions at *V*_g_ = 0 V^45,52,54^. See Supplementary Note 7 for the characterization of graphene doping level. Figure 3d illustrated the frequency response measured up to 4 kHz. The almost constant values indicate a far larger bandwidth of our device. On the other hand, since our design also leverages the conventional photoresponse at metal-graphene interfaces, the working frequency of our device is predicted to range from 10 to 500 GHz^41,43,54–56^. Despite the usage of plasmonic resonators in our device, the lifetime of plasmon is estimated to be <0.04 ps for a quality factor of 10, which is unlikely to be the limiting factor. Besides, the *RC* delay of our device, *τ*, is estimated below 1.5 ps for a given load resistance of 50 Ω^55^, which corresponds to an estimated 3 dB cutoff frequency *f*_c_ = 1/(2π*τ*) ~ 670 GHz. Figure 3e illustrates the measured photocurrent at 0^o^ polarization state as a function of the incident power. The incident power is estimated by the product of device area and power density which is obtained by measuring the total power and beam profile (Supplementary Note 8). Using linear fittings, we extract the responsivities of Device 1 at 0^o^ polarization as 16.6 mA/W and −36.3 mA/W for *V*_g_ = 0 and 80 V, respectively. With a device resistance of about 800 Ω, we obtain a peak photovoltage of −27 V/W at *V*_g_ = 80 V and a peak external efficiency of 1.1%. These values are more than two orders higher than the state-of-the-art BPVE in mid-infrared range^12^. Figure 3f shows the measured spectral density of voltage noise, which rapidly decreases when the device is operated at higher frequencies, with a knee at ~1 kHz (see Supplementary Note 9 for measurement method and raw data). In our devices with zero drain–source bias, the dark current-induced shot noise simply does not exist, and the dominant noises are 1/*f* noise and Johnson noise. Our results show that the 1/*f* noise is significant only below 1 kHz while the Johnson noise dominates at higher frequencies. Since our device can operate at speeds well beyond 1 kHz, it is not significantly affected by the large 1/*f* noise at low frequencies. We also calculate the frequency dependent noise equivalent power (NEP) by dividing the noise with responsivity, which reaches a peak value of 124 pW Hz^–1/2^. Such low NEP in our device is competing among the commercially available products (see Supplementary Note 13 for the comparison). Another figure of merit, the specific detectivity (D*), normalizes the performance to the detector size and is calculated by dividing the area of the detector by the NEP. The peak D* of Device 1 (~40 μm^2^ in area) is around 5 × 10^6^ cm Hz^1/2^ W^–1^. However, we note that the interpretation of D* should be very careful since the D* is initially proposed for those photodetectors whose noise power scales with the device area^57^, which is usually invalid for infrared detectors. In short, the D* tends to underestimate the performance of device with small active areas. To fairly compare the sensitivity of infrared detectors whose noise are insensitive to the device area, NEP or the detectivity, *D* (~1/NEP), should be preferred.

### Calibration-free detection of polarization angle

**Fig. 4:**
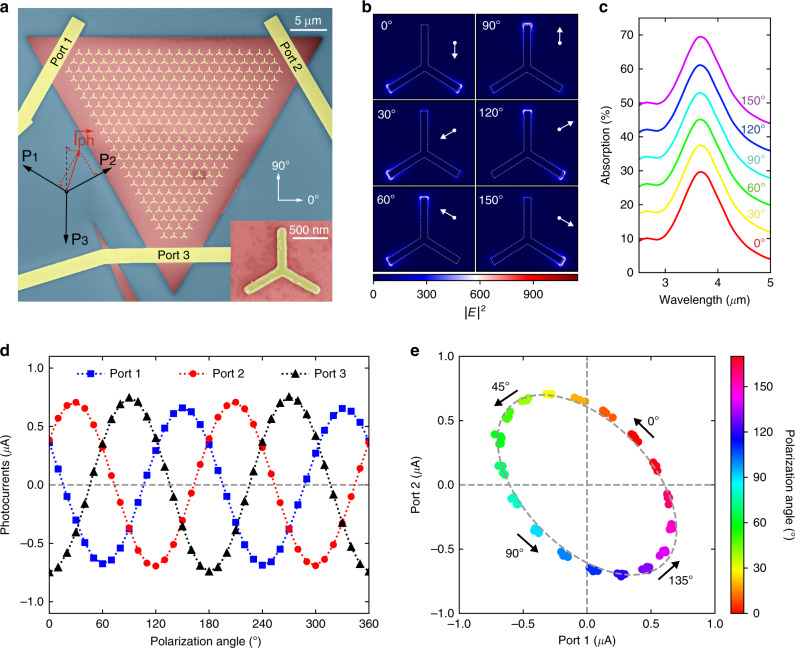
Calibration-free polarization detection. **a** SEM images of a three-port device for calibration-free polarization detection. The bottom left inset shows how the ***I***_**ph**_ is decomposed into the currents measured at the three ports. The scalar projection is valid in our case because of the three-fold rotation symmetry. Bottom right inset shows the zoom-in graph of a single triangle nanoantenna. **b** Simulated near-field distribution of the half unit cell and predicted direction of photocurrent at different polarization angles (*θ*). **c** Far-field absorption spectra of metasurface, which show no dependence on the polarization angle due to the three-fold rotation symmetry. The spectra have been shifted vertically for better clarity. **d** Measured photocurrent at the three ports as a function of polarization angles. No external bias is applied. The results can be well fitted with *P*_1_~cos(2*θ* + *π/*3), *P*_2_~cos(2*θ−π/*3), *P*_3_~cos(2*θ−π*). **e** Two-dimensional plot of *P*_1_ and *P*_2_. By sweeping the polarization angle, (*P*_1_, *P*_2_) pairs form a closed curve that fulfills the equation of an ellipse. The light intensity and polarization can then be decoupled, enabling an unambiguous detection of the polarization state using a single device.

Leveraging the design flexibility of metasurface, the proposed artificial BPVE in our work enables exotic functionalities that are unavailable in the conventional devices or even the intrinsic BPVE. As shown in Fig. 4a, we demonstrated a three-port device that can unambiguously detect the polarization angle without the calibration of incident power. Although polarization sensitive photodetectors have been reported using anisotropic materials including black phosphorous and plasmonic structures^15,16,37,58^, the mechanism is based on the scalar anisotropic absorption. As a result, the photocurrent has the same sign for all polarization states, and hence an unambiguous detection of the polarization angle remains challenging. The bottleneck can be circumvented in our device by using the artificial BPVE with vectorial photocurrent, ***I***_**ph**_. Since there are two independent variables in ***I***_**ph**_, namely, polarization angle and incident power, we need at least three ports to acquire the full information. Note that one of the three ports is not independent due to the Kirchhoff’s circuit law. Furthermore, we have intentionally designed the device to possess a three-fold rotation symmetry such that an analytical expression of the polarization dependence is available. Under such condition, the measured photocurrent at the three ports are simply the scalar projection of the ***I***_**ph**_. The detailed theoretical derivation can be found in Supplementary Note 10. As we have discussed, the ***I***_**ph**_ can be analyzed by simulating the near field of nanoantennas as illustrated in Fig. 4b. When the polarization angles increase by one cycle from 0° to 180°, the orientation of ***I***_**ph**_ is reversely rotated by 360^o^. Interestingly, despite the strong dependence of near field on the polarization, the metasurface is isotropic in terms of far-field spectra. As shown in Fig. 4c, the simulated far-field absorption spectra of the metasurface at different polarization angles are identical, which is a natural result of the three-fold rotation symmetry. This device symmetry allows us to write analytical expressions of the collected photocurrent at the three ports as (Supplementary Note 10)

5 $$\begin{array}{*{20}{c}} {\left( {\begin{array}{*{20}{c}} {P_1} \\ {P_2} \\ {P_3} \end{array}} \right) = \left( {\begin{array}{*{20}{c}} {\cos \left( {2\theta + \frac{\pi }{3}} \right)} \\ {\cos \left( {2\theta - \frac{\pi }{3}} \right)} \\ {\cos \left( {2\theta - \pi } \right)} \end{array}} \right) \cdot \left| {{\boldsymbol{I}}_{{{{\mathbf{ph}}}}}} \right|} \end{array}$$

where *θ* is the polarization angle of incident light. The magnitude of vectorial photocurrent, ***I***_**ph**_, is independent of the polarization as $\left| {{\boldsymbol{I}}_{{{{\mathbf{ph}}}}}} \right| = \alpha \left| E \right|^2$, where *α* is a scalar and *E* is the electric field of incident light. The sign of current at each port is defined as positive when it flows inward the device.

Figure 4d shows the measured photocurrent at different polarization angles but constant incident power, which are well-matched with our theoretical analysis. The slight difference between the three ports is because of fabrication errors and hence our device is not perfectly three-fold rotation symmetric. It is easy to check that Eq. (5) fulfills the Kirchhoff’s circuit law, namely, *P*_1_ + *P*_2_ + *P*_3_ = 0. Since only *P*_1_ and *P*_2_ are independent, we plot the (*P*_1_, *P*_2_) pairs in Fig. 4e with the dots colored based on their polarization angles. When the polarization angles increase, the (*P*_1_, *P*_2_) pairs move counter-clockwise along a closed curve which fulfills the equation of an ellipse

6 $$\begin{array}{*{20}{c}} {\frac{{\left( {P_2 - P_1} \right)^2}}{3} + \left( {P_2 + P_1} \right)^2 = \left| {{\boldsymbol{I}}_{{{{\mathbf{ph}}}}}} \right|^2} \end{array}$$

This curve encircles the origin and there is no intersection, which is desired for unambiguous detection. Besides, the polarization angles are calculated as

7 $$\begin{array}{*{20}{c}} {\theta = \left\{ {\begin{array}{*{20}{c}} {\frac{1}{2}\arctan \left( {\frac{1}{{\sqrt 3 }} \cdot \frac{{P_2 - P_1}}{{P_2 + P_1}}} \right),\;{\mathrm{when}}\;(P_2 + P_1) > 0} \\ {\frac{1}{2}\arctan \left( {\frac{1}{{\sqrt 3 }} \cdot \frac{{P_2 - P_1}}{{P_2 + P_1}}} \right) + \frac{\pi }{2},\;{\mathrm{when}}\;(P_2 + P_1) < 0} \end{array}} \right.} \end{array}$$

Notably, we have got rid of the incident power term in the above equation. This indicates that the polarization angle can be unambiguously determined regardless of the power of incident light, which we call a calibration-free polarization detection. Furthermore, the *θ* covers the full range of polarization angles by π. Therefore, the polarization angles can be robustly and unambiguously detected using a single device.

In most cases, the polarization state of light may not be perfectly linear but elliptical. Consequently, it is also necessary to characterize the response of photodetectors to circularly polarized (CP) light. In our device, however, the effect of CP light has been intrinsically precluded due to the three-fold rotation symmetry. Note that the CP light has circular symmetry, and hence the resultant vectorial photocurrent should be unchanged when the system is rotated by 2π/3. This is obviously impossible unless the magnitude of the photocurrent is zero. If CP dependence is desired, we should break the rotation symmetry, for example, using Device 1 or 2. Our theoretical analysis is reinforced by the numerical simulation of near-field distribution (Supplementary Note 11).

## Discussion

The zero-bias operation demonstrated here is critical for many technologies that require low power consumption such as the Internet of Things, wearable devices, and smart sensors^59^. Besides, our devices operate at room temperature, which is highly desired for the next generation infrared photodetectors^16^. Compared to the conventional optoelectronics devices based on photovoltaic or photoconducting effects, the artificial BPVE proposed in this work possesses many advantages including bulk photoresponse, cascaded output, and strong polarization dependence. Importantly, our work opens alternative possibilities for multi-functional optoelectronic devices. For example, using a designed metasurface that consists of merged T-shaped nanoantennas, we have also demonstrated a photodetector with wavelength switchable bipolar photoresponse (Supplementary Note 12). While the detectivity of our proof-of-the-concept devices is already competing among the existing methods^16^, it can be further improved by impedance matching using metal–insulator–metal metamaterial perfect absorber structure for much larger absorption^60^, replacing the substrate of graphene with boron nitride^61^, replacing metallic nanoantennas with dielectric structures for higher quality factor^62^, and reducing the contact resistance with optimized fabrication process^63^. A comparison with the state-of-the-art can be found in Supplementary Note 12. Besides, a microscopic investigation on the artificial BPVE with scanning near-field infrared nanoscopy will also help to further optimize the performance^44^. Moving forward, it will be interesting to demonstrate the artificial BPVE in visible and near-infrared range for solar energy harvesting and communications, as well as in far-infrared range for body-temperature thermal imaging applications.

In summary, we have presented metasurface-mediated BPVE-type photoresponse in mid-infrared range with advantages including zero-bias operation, high detectivity, cascaded and scalable photoresponse, calibration-free polarization detection, sub-wavelength pixel, tailorable working wavelength, and fast response. In analog to the conventional BPVE in ferroelectrics, the non-centrosymmetric structure of plasmonic nanoantenna is indispensable in our device. Conversely, the mesoscopic design of the artificial BPVE may also provide an interesting platform for the research on the intrinsic BPVE in which the inverse symmetry is broken in microscopical scale. Compared to the state-of-the-art BPVE in mid-infrared, the performance of our device is more than two orders higher. Thanks to the design flexibility of metasurface, we have also demonstrated that polarization angles can be unambiguously detected regardless of the incident power using a single device. Leveraging the compatibility of graphene with existing semiconductor fabrication lines, our work can find widespread applications in the emerging infrared technologies such as free-space communications, polarimetric imaging, and plasmon-enhanced molecule sensing.

## Methods

### Device fabrication

As the first step, graphene flakes were mechanically exfoliated from natural graphite crystals onto heavily *p*-doped silicon wafer grown with 285 nm thermal SiO_2_. Then, alignment marks were fabricated on the chips using standard electron-beam lithography (EBL, JBX-6300FS, Jeol; E-beam resist: 495 K PMMA A4; spin coating speed: 4000 rpm; EBL Dose: 1300 μC/cm^2^; Development in MIBK:IPA=1:3 for 30 s) followed with thermal deposition of 3-nm-thick Ti and 20-nm-thick Au (AJA Ebeam Evaporator) and liftoff process (submerging samples in acetone at 65 °C for 1 h). With these alignment marks, graphene flakes were then patterned to regular shapes by EBL and oxygen plasma (Vita-Mini RIE system, Femto Science. Recipe: power 20 W, O_2_ gas 20 sccm, duration 20 s). After oxygen plasma, there were many PMMA residues on the surface of graphene. These plasma-hardened PMMA residues cannot be removed by standard process, such as acetone, but can be almost completely cleaned by thermal annealing (Ar/H_2_ atmosphere at 350 °C for 6 h), which was confirmed by atomic force microscopy measurement. Then, metallic nanoantennas and contacts were patterned on graphene flakes by EBL with the assistance of alignment marks, followed with thermal deposition of 5-nm-thick Pd and 50-nm-thick Au (Lesker NANO 36 Thermal Evaporator) and liftoff process. Due to the poor adhesion of Pd to SiO_2_ wafer, another EBL and thermal deposition were conducted to fabricate electrodes (5-nm-thick Ti and 80-nm-thick Au) for probing for electrical characterization.

### Characterization

We used a quantum cascaded laser (MIRCat-1200, Daylight solutions) as the light source at 4 µm wavelength, whose polarization is linear (>100:1). A low-order half-wave plate (WPLH05M-4000, Thorlabs) designed at 4 µm was used to control the polarization angles of light. Since our device is placed horizontally, a 90° off-axis parabolic mirror (MPD149-P01, Thorlabs. Reflected focal length is 101.6 mm) was used to focus and direct light to our device. The focused light spot was measured to be elliptical with two intensity radii as 171 and 271 μm. The alignment between light spot and our device was assisted by a built-in visible laser which has been collimated with the infrared light. The *I*–*V* curves were measured with a semiconductor characterization system (Keithley 4200-SCS). In the measurement of control samples, we have always kept the drain–source orientation along the same direction, since the sign of photocurrent is critical in our work but can be flipped by exchange the drain and source electrodes. Specifically, we have always used the nearest electrode on its right side as drain and the nearest electrode on its left as source. In the measurement of time and frequency responses, an optical chopper (Stanford Research Systems, SR540) was used to modulate the light intensity. To measure the time response, the signal of our device was first amplified by a preamplifier (Stanford Research Systems, SR560) and then connected to an oscilloscope. To measure the frequency response, the drain and source ports of our device were connected directly to a lock-in amplifier (Stanford Research Systems, SR830). The noise of our device was also measured with the lock-in amplifier, in which the time constant was set to be 1 s. In the measurement of power dependence, the power of incident light was tuned by changing the current of laser and adding neutral density filters (NDIR03A, NDIR10A, NDIR20A, Thorlabs), and the total incident power was measured with a power meter (843-R, Newport). The polarization dependence was measured with the lock-in amplifier, in which opposite currents were indicated by different phases.

### Simulation

The numerical simulations in this work were carried out using the FDTD method (FDTD Solutions package from Lumerical Inc.). To simulate the optical response of nanoantennas arrays with normal incident light, we applied periodic boundary conditions at the *x*- and *y*-boundaries and perfect matched layer (PML) condition at the *z*-boundaries. Mesh spacing was 10 nm in all dimensions.

## Supplementary information

Supplementary Information

## Data Availability

All technical details for producing the figures are enclosed in the supplementary information. Data are available from the corresponding authors C.-W.Q. or C.L. upon request.
