# Supplementary material for: Zero-bias mid-infrared graphene photodetectors with bulk photoresponse and calibration-free polarization detection
Source: Nat Commun. 2020 Dec 17;11:6404. doi: 10.1038/s41467-020-20115-1 (PMC7747747; doi:10.1038/s41467-020-20115-1)
Supplement: Supplementary file 1 — Supplementary Information [file 41467_2020_20115_MOESM1_ESM.pdf]

Supplementary Information

Zero-bias Mid-infrared Graphene Photodetectors with Bulk Photoresponse and Calibration-free Polarization Detection

Jingxuan Wei[1,2], Ying Li[1], Lin Wang[1], Wugang Liao[1], Bowei Dong[1,2], Cheng Xu[1,2], Chunxiang Zhu[1], Kah-Wee Ang[1], Cheng-Wei Qiu[1,*], and Chengkuo Lee[1,2,*]

[1] Department of Electrical and Computer Engineering, National University of Singapore, 117583, Singapore

[2] Center for Intelligent Sensors and MEMS, National University of Singapore, 117608, Singapore

[*] E-mail: (C. L.) elelc@nus.edu.sg and (C.W. Q.) chengwei.qiu@nus.edu.sg

Supplementary Notes 1-13

Supplementary Information References

Supplementary Note 1. Seebeck coefficient of graphene

As a bipolar semiconductor with zero-bandgap, the Seebeck coefficient of graphene (S) can be estimated through Mott relation[1]

$$S = -\frac{\pi^2 k_B^2 T}{3e}\frac{1}{\sigma}\frac{\partial \sigma}{\partial \phi} \tag{1}$$

where Φ, σ, k_B, T and e are chemical potential, electrical conductance, Boltzmann constant, temperature and elementary charge, respectively. Note that the conductance σ of graphene is a function of its chemical potential (Φ).[2]

$$\sigma = \frac{W}{L}\left(n_0 + \frac{\phi^2}{\pi(hv)^2}\right)e\mu \tag{2}$$

where W and L are the width and length of the graphene device. n_0 is the residual carrier concentration. h, v and μ are Planck constant, Fermi velocity and mobility, respectively. The Fermi velocity of graphene is about 10^6 m/s.[1] By substituting Supplementary Equation (2) into Supplementary Equation (1), we can obtain the expression of S, which is directly dependent on Φ:

$$S = -\frac{2\pi^2 k_B^2 T}{3e}\frac{\phi}{\pi n_0 (hv)^2 + \phi^2} \tag{3}$$

Therefore, we can plot the Seebeck coefficient of graphene as a function of its chemical potential as Supplementary Figure 1. Here we have assumed a typical value of n_0 as 2×10^{12} cm^{-2} and room temperature operation (T=300K). As we can see from the graph, the Seebeck coefficient of graphene is non-monotonous and changes its sign for *p*- and *n*-type doping. Besides, due to the Fermi level pinning, the graphene covered by metals shows fixed Seebeck coefficients (S_{mg}). In contrast, since the chemical potential of uncovered graphene can be tuned electrically via gate voltage, its Seebeck coefficient (S_g) can be moved along the curves. The resultant difference of Seebeck coefficients, $\Delta S = S_{mg} - S_g$, controls the sign and amplitude of photocurrents generated at metal-graphene interfaces.

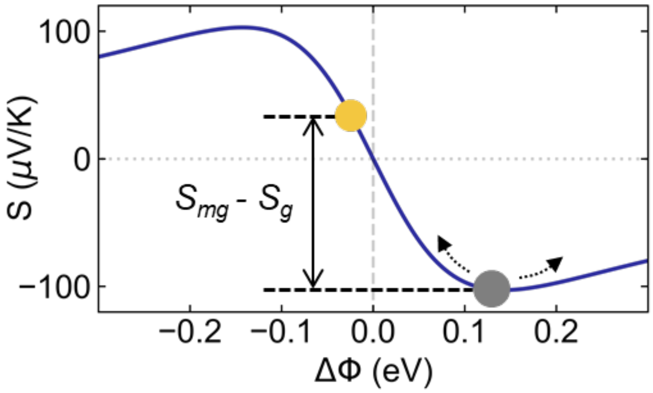

Supplementary Figure 1 | Seebeck coefficient of graphene (*S*) as a function of the chemical potential ($\Delta\Phi$). The yellow dot represents the graphene covered with metal contact, while the grey dot denotes the uncovered graphene. Unlike the covered graphene whose chemical potential is pinned, that of the uncovered graphene can be tuned electrically, so that the $\Delta S = S_{mg} - S_g$ is also tunable.

Supplementary Note 2. Wavelength dependent plasmonic resonance

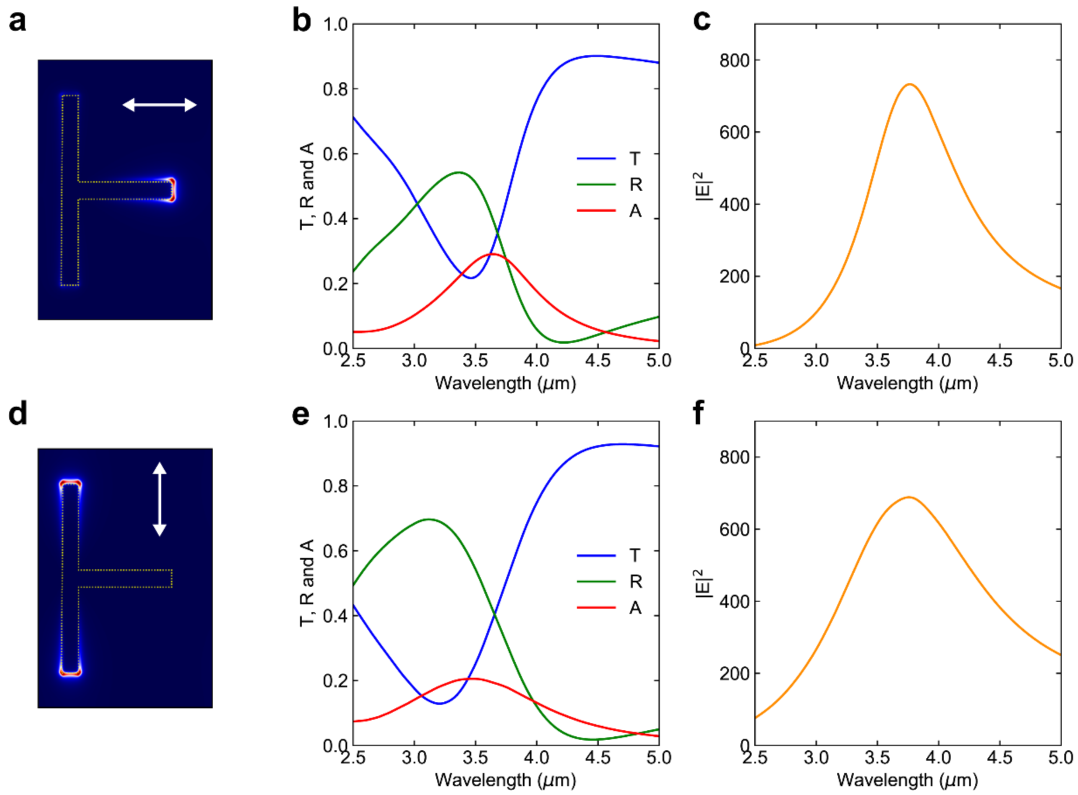

Supplementary Figure 2 | Plasmonic resonance of T-shaped nanoantennas. **a**, Illustrated near-field distribution of T-shaped nanoantennas with horizontally polarized incident light. **b-c**, Simulated wavelength dependent (b) far-field spectra and (c) peak near-field enhancement. **d**, Illustrated near-field distribution of T-shaped nanoantennas with vertically polarized incident light. **e-f**, Simulated wavelength dependent (e) far-field spectra and (f) peak near-field enhancement. *T*, *R* and *A* represent the transmission, reflection and absorption spectra, respectively.

Supplementary Note 3. Raman spectroscopy

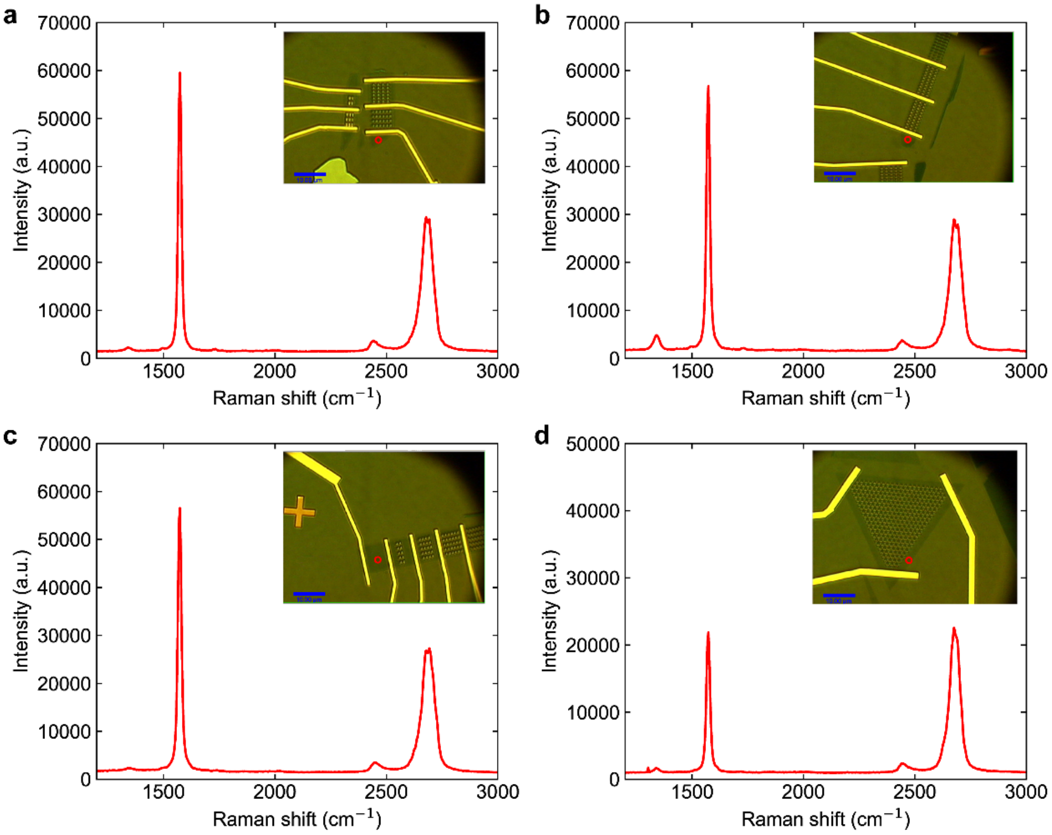

Supplementary Figure 3 | Raman spectra. **a-d**, Measured Raman spectra with a 532 nm laser of the devices shown in Supplementary Figures 1, 2a, 2b, and 4, respectively. The insets show the optical images with scale bars of 10 μm. The hollow red dots represent the measurement location. The small ratios of 2D band signals (~2680 cm^{-1}) to G band signals (~1574 cm^{-1}) suggest the thickness of graphene flakes in our device are bilayer for the three-port device and a few layers for other devices[3]. Besides, the weak peaks of D band (~1350 cm^{-1}) suggest a relatively clean surface of graphene[4].

Supplementary Note 4. Atomic force microscopy (AFM) measurement

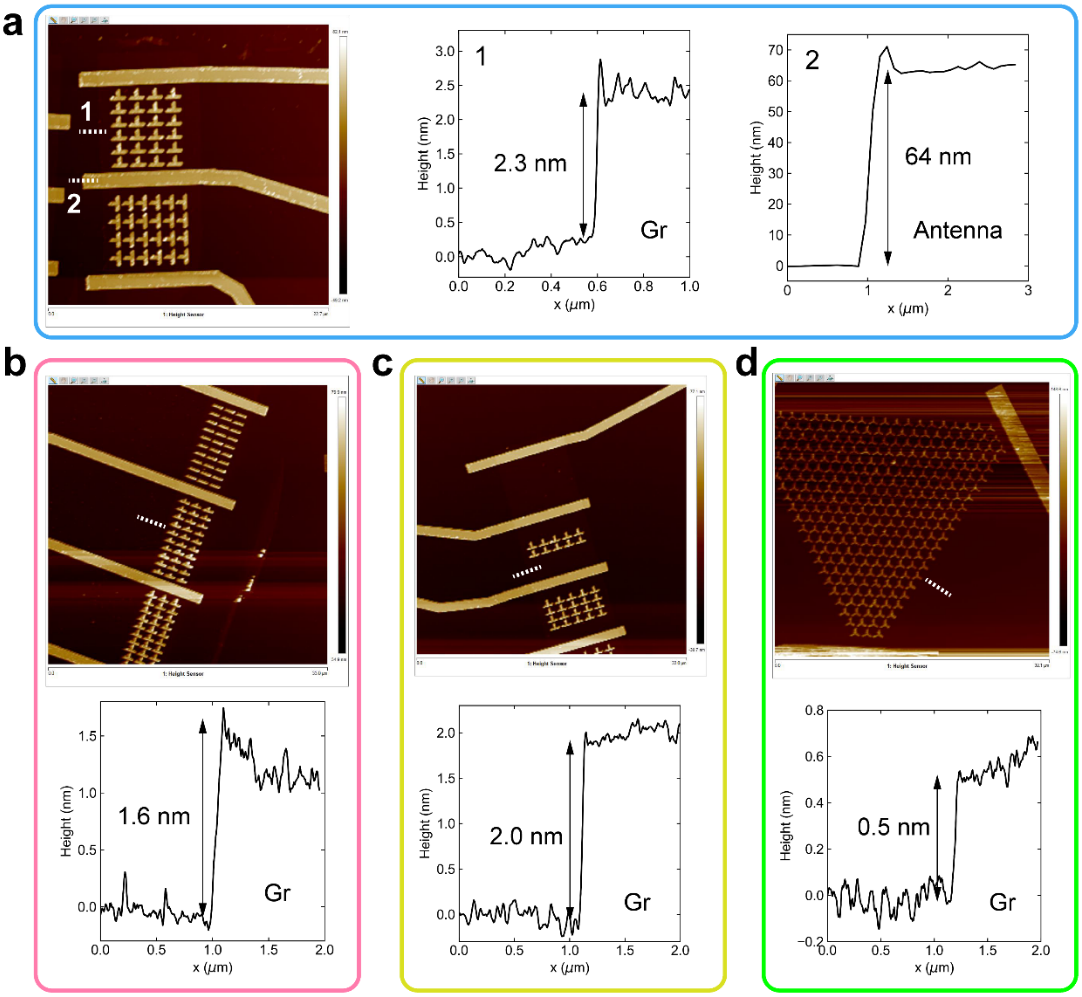

Supplementary Figure 4 | AFM measurement. **a-d**, Measured AFM images of the devices shown in Supplementary Figures 1, 2a, 2b, and 4, respectively. The white dotted lines indicate the cross section to measure the thickness of graphene and nanoantennas. The estimated thickness of graphene is matched with that of Raman spectra[5].

Supplementary Note 5. Photocurrent generation from nanoantennas with different degrees of asymmetry

To investigate the dependence of photocurrents on the degree of asymmetry, we simulated the near-field distribution of nanoantennas with different lengths of horizontal bars (L_h), as shown in Supplementary Figure 5. At 0° polarization angle, the reduction of L_h leads to the vanishing plasmonic resonance. On the other hand, at 90° polarization angle, the length of L_h has little effect on the plasmonic resonance but will affect the conductance profile. Since the resonance wavelength is highly sensitive to the length of electrical length of nanoantennas, the two different mechanisms may explain the more sensitive dependence of photoresponse on the L_h at 0°.

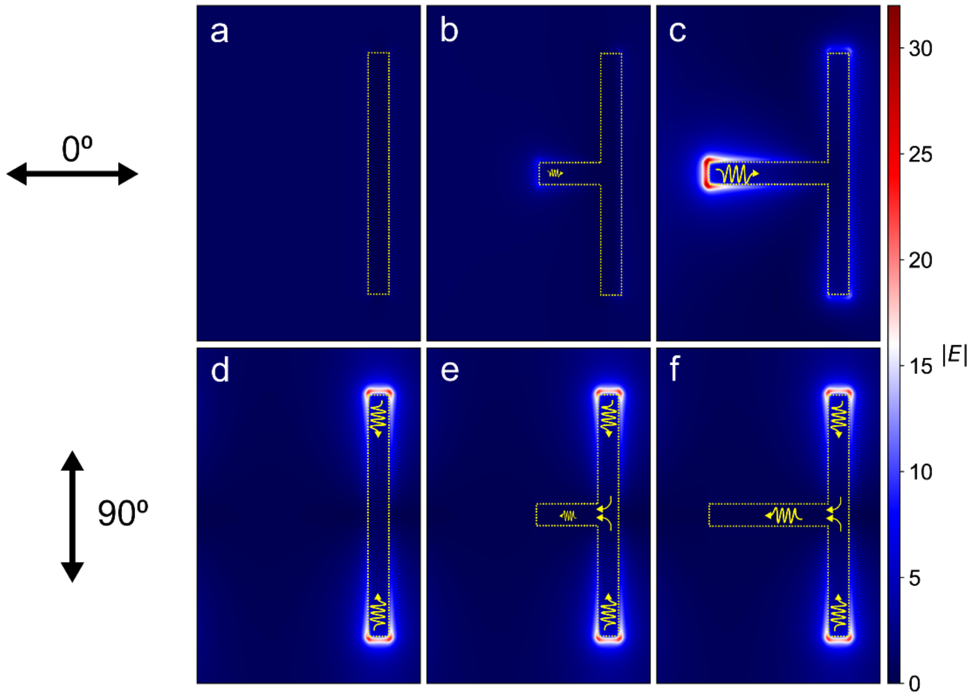

Supplementary Figure 5 | Simulated near-field distribution of nanoantennas and the resultant flow of photocarriers. 0° and 90° indicate the polarization angles of incident plane wave. Dotted lines are the edges of nanoantennas. Yellow wave arrows represent the flow of photocarriers.

Supplementary Note 6. Dependence of photovoltages on the number of nanoantennas

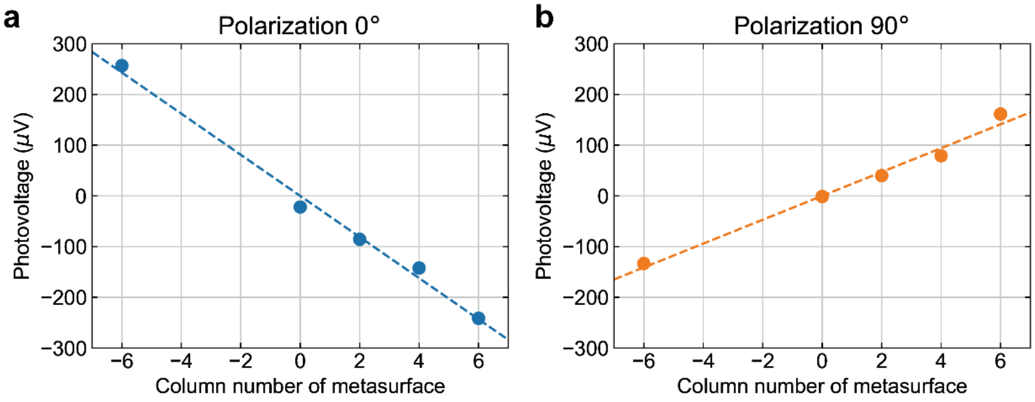

Supplementary Figure 6 | Fitting of the measured photovoltages versus the number of nanoantennas. The negative value of column number indicates the reversed orientation of nanoantennas. The good linear fitting suggests a cascaded photoresponse in our device.

Supplementary Note 7. Gate controlled doping in graphene

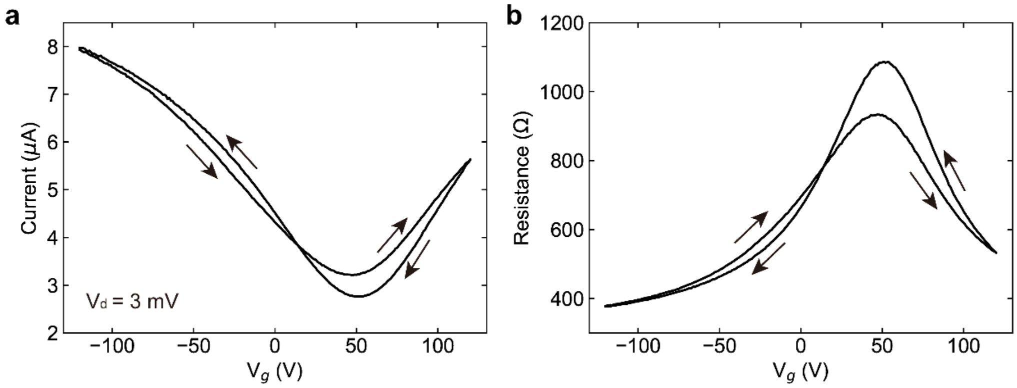

Supplementary Figure 7 | Characterization of doping level of graphene transistor. a, Measured I_d-V_g curves at dark condition. The applied drain-source bias is 3 mV. Black arrows indicate the sweeping direction. **b**, Measured resistance versus V_g. The neutral charge point (NCP) is achieved at V_g = 50 V. Hence, graphene is *p*-type doped at lower V_g and *n*-type at higher V_g. At zero bias condition (V_g=0 V), the graphene is lightly *p*-doped.

Supplementary Note 8. Beam profile

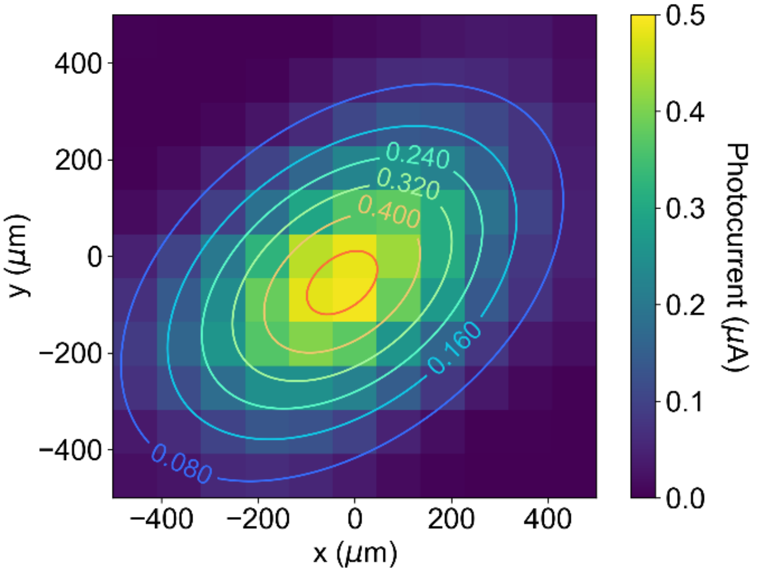

Supplementary Figure 8 | Photocurrent mapping via moving the device location. Due to the small size of our device (~10 μm), it can be used as a point probe to measure the beam profile by moving the device stage. In total, 11 by 11 points are measured with a spatial step of 100 μm. As we can see in the responsivity measurement, the photocurrents are almost linear to the incident power. Hence, we can directly use the photocurrent map to extract the beam profile, which is elliptical with the two axes radii ($1/e^2$ intensity) as 271 and 171 μm.

By moving the device, we obtain the photocurrent map and hence the beam profile. The measured beam profile can be fitted with Gaussian beam as

$$I(r) = I_0 \exp\left(\frac{-2r^2}{r_1 r_2}\right) \tag{4}$$

where I is the intensity, and I_0 is the intensity at the center of the beam. r_1 and r_2 are the radii along the two axes, which are extracted as 271 and 171 μm. The peak intensity I_0 is related to the total power of Gaussian beam[6]

$$I_0 = \frac{2P_0}{\pi r_1 r_2} \tag{5}$$

In our experiment, we have always optimized the photocurrent by moving the device to the center of beam. Since the area of our device (S) is small, we can consider that the power density on our device is I_0, and hence the incident power onto our device is $P = I_0 * S$.

Supplementary Note 9. Noise measurement

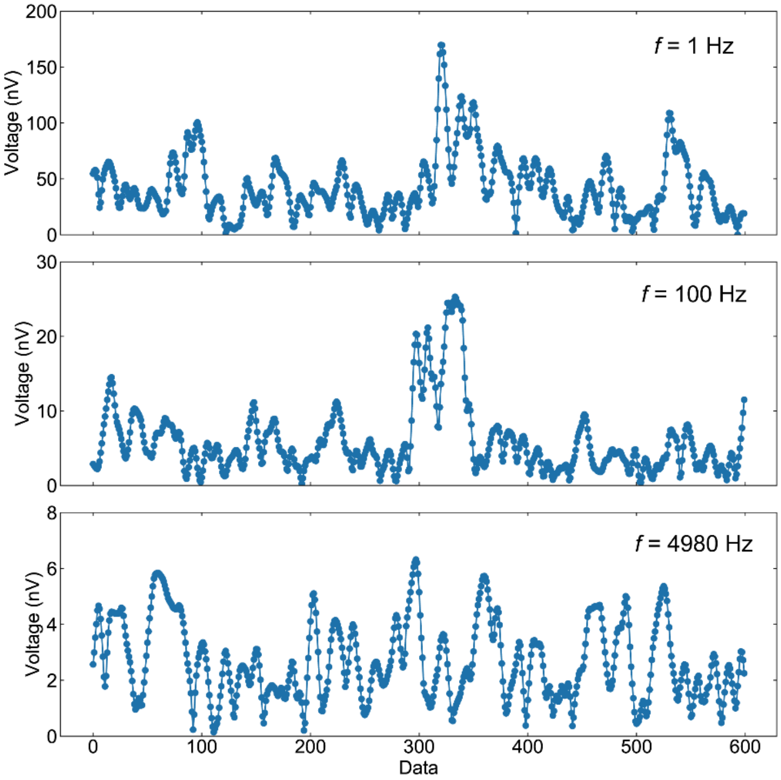

Supplementary Figure 9 | Noise measurement with a Lock-in amplifier. The voltages are measured by directly plugging the drain and source electrodes to a Lock-in amplifier. The frequency is set internally. The data were collected with a time constant of 1s.

The noise voltage was measured with a lock-in amplifier. As shown Supplementary Figure S9, the magnitude of voltages fluctuates along with time. The data were collected at a time constant of 1s. The noise spectra are calculated via

$$V_{noise} = \sqrt{\overline{V_n^2}} \tag{6}$$

The noise current of our device is related with the noise voltage as $I_{noise} = V_{noise}/R$, where R is the device resistance.

Supplementary Note 10. Theoretical analysis of three-port device

In this section, we provide a theoretical description of the polarization dependence of the three-port photodetector. To start with, we consider the plasmon resonance at 0° polarization as shown in Supplementary Figure 10a. At such condition, the incident light will excite oscillating charges at the bottom tips of the triangle nanoantennas. Due to the mirror symmetry, there will be no charges on the top tip. The above analysis is confirmed by the near-field simulation as Figure 4b (main manuscript). Similarly, Supplementary Figure 10b and Supplementary Figure 10c show the resonance modes at 120° and 240° polarization angles.

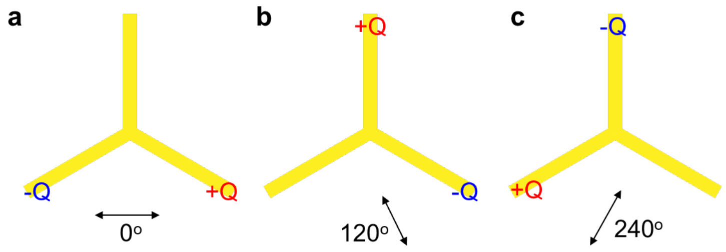

Supplementary Figure 10 | Resonance modes of nanoantennas at 0°, 120° and 240° polarization angles. +Q and −Q denotes the oscillating charges in plasmonic mode, with the "+" and "–" signs illustrating the relative phase to incident light.

Then, we consider the resonance mode at 90° polarization. Mathematically, the incident light at 90° polarization can be decomposed into two components at 60° and 120° polarization as shown in Supplementary Figure 11a. Therefore, the resonance mode is a

superposition of two basic modes as shown in Supplementary Figure 11b. Note that there is a phase difference of π between the light at 60° and 240°.

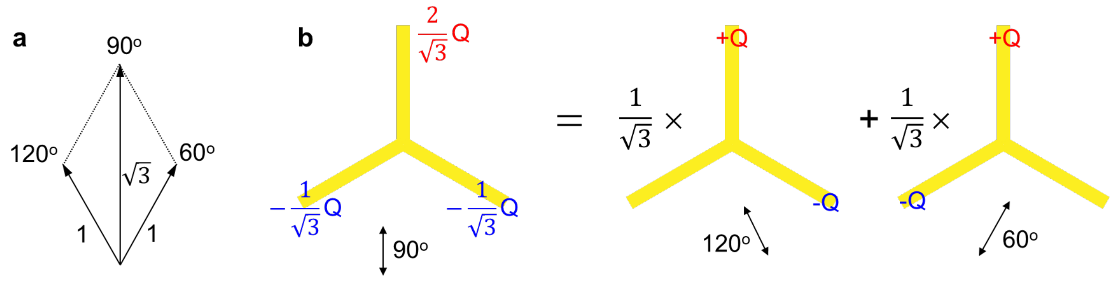

Supplementary Figure 11 | Resonance modes of nanoantennas at 90° polarization angle. a, Composition of the polarized light. **b**, Calculation of the resonance mode at 90° based on two basic modes.

With the resonance modes at two orthogonal angles, namely 0° and 60°, we can write the expression of resonance modes at arbitrary polarization angle

$$\begin{pmatrix} Q_1 \\ Q_2 \\ Q_3 \end{pmatrix} = \cos\theta \cdot \begin{pmatrix} 0 \\ -Q \\ Q \end{pmatrix} + \sin\theta \cdot \begin{pmatrix} \frac{2}{\sqrt{3}}Q \\ -\frac{1}{\sqrt{3}}Q \\ -\frac{1}{\sqrt{3}}Q \end{pmatrix}$$

$$= \begin{pmatrix} \frac{2}{\sqrt{3}}\sin\theta \\ -\cos\theta - \frac{1}{\sqrt{3}}\sin\theta \\ \cos\theta - \frac{1}{\sqrt{3}}\sin\theta \end{pmatrix} \cdot Q \quad (7)$$

where Q_1, Q_2 and Q_3 are the oscillating charges at the top, bottom left and bottom right ends of the triangle nanoantennas. According to Gaussian law, the near-field amplitude at the three tips are

$$\begin{pmatrix} E_1 \\ E_2 \\ E_3 \end{pmatrix} \sim \begin{pmatrix} Q_1 \\ Q_2 \\ Q_3 \end{pmatrix} \quad (8)$$

With the information on the electric field, we then derive the polarization dependence of photocurrents. We first consider the contribution from the top tip of nanoantennas. When photocarriers are generated and separated at the top tip, a net photocurrent flow upwards. For the photocurrents generated at the bottom left and bottom right tips, the orientation of the photocurrents are rotated by 120° and 240°. The generated photocurrents are of course proportional to the light intensity. Therefore, the vectorial photocurrent can be written as

$$\overrightarrow{I_{ph}} = \alpha|E_1|^2 \cdot \overrightarrow{n_T} + \alpha|E_2|^2 \cdot \overrightarrow{n_{BL}} + \alpha|E_3|^2 \cdot \overrightarrow{n_{BR}}$$

$$= \alpha|E_1|^2 \cdot \begin{pmatrix} 0 \\ 1 \end{pmatrix} + \alpha|E_2|^2 \cdot \begin{pmatrix} -\frac{\sqrt{3}}{2} \\ -\frac{1}{2} \end{pmatrix} + \alpha|E_3|^2 \cdot \begin{pmatrix} \frac{\sqrt{3}}{2} \\ -\frac{1}{2} \end{pmatrix}$$

$$= \alpha|E_0|^2$$

$$\cdot \begin{pmatrix} -\frac{\sqrt{3}}{2} \cdot \left(\cos\theta + \frac{1}{\sqrt{3}}\sin\theta\right)^2 + \frac{\sqrt{3}}{2} \cdot \left(\cos\theta - \frac{1}{\sqrt{3}}\sin\theta\right)^2 \\ \left(\frac{2}{\sqrt{3}}\sin\theta\right)^2 - \frac{1}{2} \cdot \left(\cos\theta + \frac{1}{\sqrt{3}}\sin\theta\right)^2 - \frac{1}{2} \cdot \left(\cos\theta - \frac{1}{\sqrt{3}}\sin\theta\right)^2 \end{pmatrix}$$

$$= \alpha|E_0|^2 \cdot \begin{pmatrix} -\sin 2\theta \\ -\cos 2\theta \end{pmatrix} \sim \begin{pmatrix} cos\left(-\frac{\pi}{2} - 2\theta\right) \\ sin\left(-\frac{\pi}{2} - 2\theta\right) \end{pmatrix} \quad (9)$$

where α is the parameter that relates the photocurrents with the field intensity. E_0 is a fitting factor. $\overrightarrow{n_T}$, $\overrightarrow{n_{BL}}$ and $\overrightarrow{n_{BR}}$ are the unit vectors in the normal direction of metal-graphene interfaces at the top, bottom left, and bottom right tips of triangle nanoantennas. The angle of the vectorial photocurrents at θ polarization angle is $-\pi/2-2\theta$.

We then consider the photocurrents that are measured at the three ports. We also start from a unit photocurrent generated at the top tip of triangle nanoantennas. Easily, we will collect photocurrents (P_1, P_2, P_3) ~ (-1/2, -1/2, 1). For the bottom left tip, (P_1, P_2, P_3) ~ (-1/2, 1, -1/2). For the bottom right tip, (P_1, P_2, P_3) ~ (1, -1/2, -1/2). Thus, the polarization dependent photocurrents at the three ports are

$$\begin{pmatrix} P_1 \\ P_2 \\ P_3 \end{pmatrix} \sim \left(\frac{2}{\sqrt{3}}\sin\theta\right)^2 \begin{pmatrix} -\frac{1}{2} \\ -\frac{1}{2} \\ 1 \end{pmatrix} + \left(\cos\theta + \frac{1}{\sqrt{3}}\sin\theta\right)^2 \begin{pmatrix} -\frac{1}{2} \\ 1 \\ -\frac{1}{2} \end{pmatrix} + \left(\cos\theta - \frac{1}{\sqrt{3}}\sin\theta\right)^2 \begin{pmatrix} 1 \\ -\frac{1}{2} \\ -\frac{1}{2} \end{pmatrix}$$

$$= \begin{pmatrix} \cos\left(2\theta + \frac{\pi}{3}\right) \\ \cos\left(2\theta - \frac{\pi}{3}\right) \\ \cos(2\theta - \pi) \end{pmatrix} \quad (10)$$

Interestingly, the above results show that the P_1, P_2, P_3 are the scalar projections of $\overrightarrow{I_{ph}}$ onto the unit vectors with angles of 150°, 270° and 30°, respectively. Since the photocurrents are defined as positive for outward flow, the projection shown in the inset of Figure 4a (main manuscript) is valid.

Supplementary Note 11. Photocurrents at circular polarization states

In the BPVE, the shift currents only emerge from linearly polarized light. When the incident light is circularly polarized, however, another mechanism may also lead to short-circuit current, namely, the injection current[7,8]. This is also usually referred to as circular photogalvanic effect. In Supplementary Figure 12, we investigate the possible photoresponse of the T-shaped nanoantennas under circular polarized illumination. It is dependent on whether the circularly polarized light will excite asymmetric near field, which is related to the spectral detuning between the two eigenmodes excited by orthogonal linearly polarized light. As a result, we anticipate a negligible photoresponse for nanoantennas with L_h = 600 nm, but non-zero and opposite photoresponse for nanoantennas with L_h = 500 nm and 700 nm.

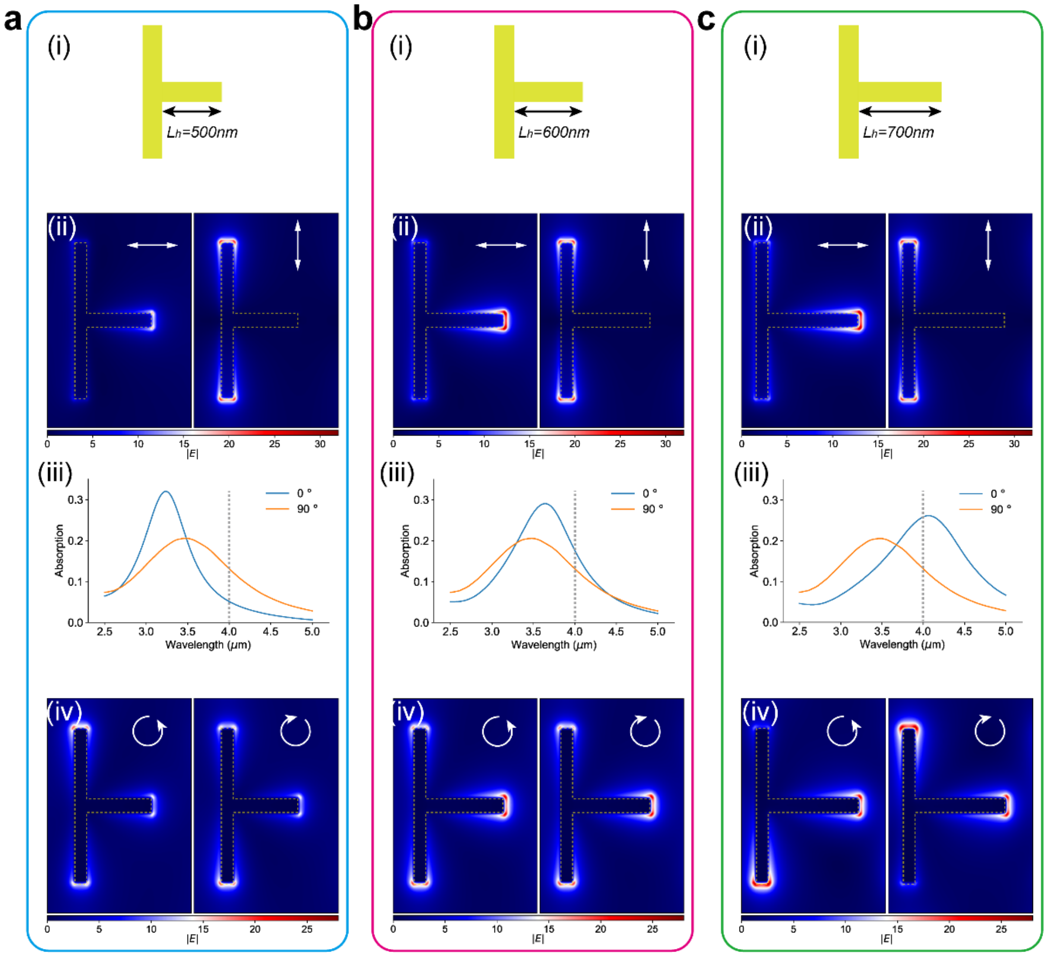

Supplementary Figure 12 | Studies on the near-field distribution of T-shaped nanoantennas with different L_h under circular polarized illumination. The Circular polarized light can be regarded as a superposition of two orthogonally linearly polarized components with a phase difference of π/2. When the L_h changes from (a) 500 nm to (c) 700 nm, the plasmonic resonances at 0° and 90° polarization are also altered relatively. Notably, the resonance wavelength is shifted for 0° polarization, which leads to a tunable phase delay between two plasmonic modes excited by 0° and 90° polarization light. As a result, the phase difference of the excited plasmonic resonances at 0° and 90° polarization may not be π/2, leading to constructive or destructive interferences. The resultant asymmetric field profile will of course lead to photocurrents in our device. The anti-clockwise/clockwise arrows indicate left/right-handed circularly polarized wave as defined from the point of view of the source.

Furthermore, we consider the circular dependence of photoresponse for our three-port device as shown in Figure 4 (main manuscript). Supplementary Figure 13 shows the near-field profile under illumination at circular polarization states. Obviously, the near field also possesses a three-fold rotation symmetry as our theoretical analysis. Therefore, we anticipate that the photocurrents in such devices will vanish.

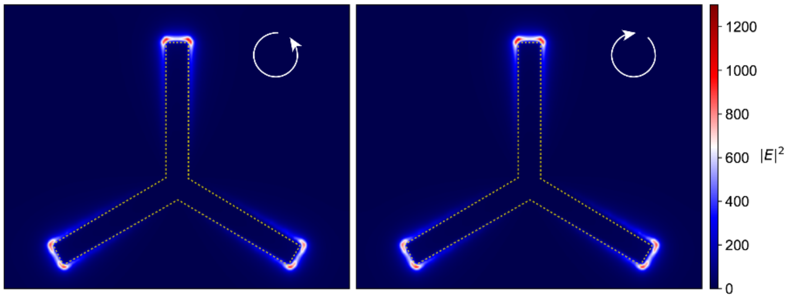

Supplementary Figure 13 | Studies on the near-field distribution of triangle nanoantennas under circular polarized illumination.

Supplementary Note 12. Wavelength switchable bipolar photoresponse

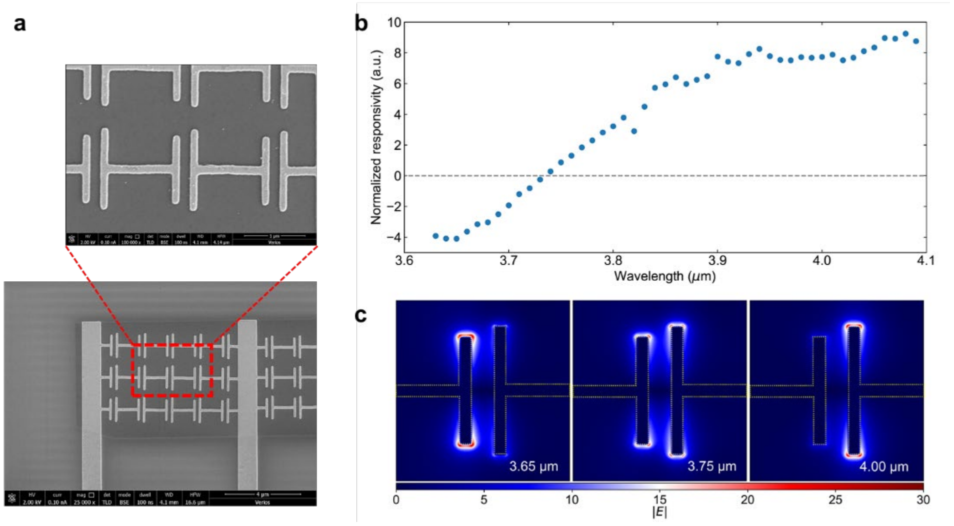

Supplementary Figure 14 | Wavelength switchable bipolar photoresponse using non-Hermitian nanoantennas. a, SEM images of the fabricated devices. The scale bars are 4 μm and 1 μm in the bottom and top images. **b**, Measured photovoltages with vertical polarization angle of incident light. The sign is flipped around 3.73 μm. **c**, Simulated near-field distribution of nanoantennas at different wavelengths. The very asymmetric field is due to the non-Hermitian coupling between the nanoantennas with slightly different lengths[9].

Supplementary Note 13. Comparison with previous works

**Supplementary Table 1 | Performance parameters**					
**Description**	**Mechanism**	**Zero bias?**	**λ (μm)**	**Responsivity**	**NEP ($nW/Hz^{1/2}$)**
Graphene–metal interfaces[10,11]	PTE & PV	Y	0.4-1.55	<1.5 mA/W	-
Graphene p–n junction[12]	PTE & PV	Y	0.53	0.75 mA/W	-
Chip integrated Graphene photodetector[13,14]	PTE & PV	Y	1.45-1.59	16-50 mA/W	-
Tip contacted $BaTiO_3$[15]	BPVE	Y	0.4	605 mA/W	-
Flexo-photovoltaics[16]	BPVE	Y	0.4	-	-
WS_2 nanotubes[17]	BPVE	Y	0.53-0.73	10~200 mA/W	-
Metasurface-pyroelectric AlN detector[18]	PyE	Y	0.65-2	0.18 V/W	679 (1 kHz)
BP/MoS_2 photodiode[19]	PV	Y	2-3.8	900 mA/W	3.2×10^{-4} (N.A.)
Metamaterial integrated quantum well[20]	PC	N	9	200 mA/W at T_{room}	0.4 (1,059 Hz)
Hybrid nanoparticle-graphene[21]	PC	N	4.5-10	150 A/W	6.7×10^{-3} (Calc)
Graphene pyroelectric bolometer[22]	PyE & PC	N	7.5-10	0.23 mA/W	500 (36 Hz)
Graphene nanoribbons and nanodisks[23]	PC	N	12.2	16 mA/W	1.3 (> 1kHz)
Graphene-Based Thermopile[24]	PTE	Y	10.6	7~9 V/W	0.013 (Calc)
b-AsP/MoS_2[25]	PV	Y	3-5	220 mA/W	2.4×10^{-4} (Calc)
Weyl semimetal[26]	BPVE	Y	10.6	0.067 mA/W	-
Metasurface-mediated graphene photodetector (this work)	Artificial BPVE	Y	4	36.3 mA/W 27 V/W	0.124 (> 1kHz)

*The abbreviations of mechanisms represent photo-thermoelectric (PTE), photovoltaic (PV), pyroelectric (PyE), photoconducting (PC) and bulk photovoltaic (BPVE) effects. λ denotes the working range of wavelength. NEP means noise equivalent power.

** The modulation frequency used for NEP measurement is shown in the bracket. "Calc" indicates that the value is derived without direct measurement of either the frequency dependent responsivity or the frequency dependent noise.

In Supplementary Table 2, we compare our device with the products available in the market. Product 1-7 are microbolometers from Thorlabs; Product 8 is thermopile from Hamamatsu; Product 9 is microbolometer from Leonardo DRS. Noise equivalent power (*NEP*) characterizes the lowest power that a device can detect, and thus it is a good figure of merit to compare sensitivities. From the table, the *NEP* of our device is lower than most of other devices. Note that there is still space to improve the performance by using cleaner graphene and reducing contamination during fabrication process. Therefore, we believe that our device is competing and can be useful for practical mid-IR applications.

Supplementary Table 2: Comparison with the commercially available devices

No.	Item	Materials	Wavelength Range (μm)	Maximum bandwidth	NEP (nW/Hz$^{1/2}$)	Biased device?	Price
**1**	PDA10PT	InAsSb	1.0 - 5.8	1600 kHz	0.15	Yes	$4,227
**2**	PDA07P2	InAsSb	2.7 - 5.3	9 MHz	0.1	Yes	$528
**3**	PDA20H	PbSe	1.5 - 4.8	10 kHz	0.15	Yes	$484
**4**	PDA10JT	HgCdTe	2.0 - 5.4	160 kHz	0.18	Yes	$4,449
**5**	PDAVJ5	HgCdTe	2.7 - 5.0	1 MHz	0.014	Yes	$1,934
**6**	PDAVJ8	HgCdTe	2.0 - 8.0	100 MHz	0.17	Yes	$3,172
**7**	PDAVJ10	HgCdTe	2.0 - 10.6	100 MHz	0.21	Yes	$3,734
**8**	T11262-01	Si	3.0-5.0	50 Hz	0.9	No	-
**9**	U3510	VOx	8 - 14	> 56 Hz	-	Yes	-
**10**	**Our work**	**Graphene**	**Adjustable**	**>10 GHz**	**0.12**	**No**	**-**

Supplementary References

1. Zuev, Y. M., Chang, W. & Kim, P. Thermoelectric and Magnetothermoelectric Transport Measurements of Graphene. *Phys. Rev. Lett.* **102**, 096807 (2009).

2. Kim, S. *et al.* Realization of a high mobility dual-gated graphene field-effect transistor with Al2O3 dielectric. *Appl. Phys. Lett.* **94**, 2007–2010 (2009).

3. Liu, Y., Liu, Z., Lew, W. & Wang, Q. Temperature dependence of the electrical transport properties in few-layer graphene interconnects. *Nanoscale Res. Lett.* **8**, 335 (2013).

4. Lin, L. *et al.* Towards super-clean graphene. *Nat. Commun.* **10**, 1–7 (2019).

5. Shearer, C. J., Slattery, A. D., Stapleton, A. J., Shapter, J. G. & Gibson, C. T. Accurate thickness measurement of graphene. *Nanotechnology* **27**, (2016).

6. B. Saleh, M. Teich (Wiley, 2007) WW. Fundamentals of Photonics 2nd ed. **1**, 1370 (2007).

7. Dhara, S., Mele, E. J. & Agarwal, R. Voltage-tunable circular photogalvanic effect in silicon nanowires. *Science* **349**, 726–9 (2015).

8. Pan, Y. *et al.* Helicity dependent photocurrent in electrically gated $(Bi_{1-x}Sb_x)_2Te_3$ thin films. *Nat. Commun.* **8**, 1037 (2017).

9. Zhang, S. *et al.* Anti-Hermitian Plasmon Coupling of an Array of Gold Thin-Film Antennas for Controlling Light at the Nanoscale. *Phys. Rev. Lett.* **109**, 193902 (2012).

10. Xia, F., Mueller, T., Lin, Y., Valdes-Garcia, A. & Avouris, P. Ultrafast graphene photodetector. *Nat. Nanotechnol.* **4**, 839–843 (2009).

11. Mueller, T., Xia, F. & Avouris, P. Graphene photodetectors for high-speed optical communications. *Nat. Photonics* **4**, 297–301 (2010).

12. Gabor, N. M. *et al.* Hot carrier-assisted intrinsic photoresponse in graphene. *Science* **334**, 648–52 (2011).

13. Wang, X., Cheng, Z., Xu, K., Tsang, H. K. & Xu, J. Bin. High-responsivity graphene/silicon-heterostructure waveguide photodetectors. *Nat. Photonics* **7**, 888–891 (2013).

14. Pospischil, A. *et al.* CMOS-compatible graphene photodetector covering all optical communication bands. *Nat. Photonics* **7**, 892–896 (2013).

15. Spanier, J. E. *et al.* Power conversion efficiency exceeding the Shockley–Queisser limit in a ferroelectric insulator. *Nat. Photonics* **10**, 611–616 (2016).

16. Yang, M., Kim, D. J. & Alexe, M. Flexo-photovoltaic effect. *Science* **360**, 904–907 (2018).

17. Zhang, Y. J. *et al.* Enhanced intrinsic photovoltaic effect in tungsten disulfide

nanotubes. *Nature* **570**, 349–353 (2019).

18. Stewart, J. W., Vella, J. H., Li, W., Fan, S. & Mikkelsen, M. H. Ultrafast pyroelectric photodetection with on-chip spectral filters. *Nat. Mater.* **19**, 158–162 (2020).
19. Bullock, J. *et al.* Polarization-resolved black phosphorus/molybdenum disulfide mid-wave infrared photodiodes with high detectivity at room temperature. *Nat. Photonics* **12**, 601–607 (2018).
20. Palaferri, D. *et al.* Room-temperature nine-µm-wavelength photodetectors and GHz-frequency heterodyne receivers. *Nature* **556**, 85–88 (2018).
21. Yu, X. *et al.* Narrow bandgap oxide nanoparticles coupled with graphene for high performance mid-infrared photodetection. *Nat. Commun.* **9**, 4299 (2018).
22. Sassi, U. *et al.* Graphene-based mid-infrared room-temperature pyroelectric bolometers with ultrahigh temperature coefficient of resistance. *Nat. Commun.* **8**, 14311 (2017).
23. Guo, Q. *et al.* Efficient electrical detection of mid-infrared graphene plasmons at room temperature. *Nat. Mater.* **17**, 986–992 (2018).
24. Hsu, A. L. *et al.* Graphene-Based Thermopile for Thermal Imaging Applications. *Nano Lett.* **15**, 7211–7216 (2015).
25. Long, M. *et al.* Room temperature high-detectivity mid-infrared photodetectors based on black arsenic phosphorus. *Sci. Adv.* **3**, e1700589 (2017).
26. Osterhoudt, G. B. *et al.* Colossal mid-infrared bulk photovoltaic effect in a type-I Weyl semimetal. *Nat. Mater.* **18**, 471–475 (2019).
